# A compact LED-based projection microstereolithography for producing 3D microstructures

**DOI:** 10.1038/s41598-019-56044-3

**Published:** 2019-12-23

**Authors:** Ebrahim Behroodi, Hamid Latifi, Farhood Najafi

**Affiliations:** 1 Laser and Plasma Research Institute, Shahid Beheshti University, Tehran, 1983963113 Iran; 2 Department of Physics, Shahid Beheshti University, Tehran, 1983963113 Iran; 3 Department of Resin and Additives, Institute for Color Science and Technology, Tehran, 16765-654 Iran

**Keywords:** Applied optics, Biotechnology, Chemistry, Engineering, Optics and photonics

## Abstract

Projection microstereolithography (PµSL) is a promising additive manufacturing technique due to its low cost, accuracy, speed, and also the diversity of the materials that it can use. Recently it has shown great potentials in various applications such as microfluidics, tissue engineering, micro-optics, biomedical microdevices, and so on. However, studies on PµSL are still ongoing in terms of the quality and accuracy of the construction process, which particularly affect the fabrication of complex 3D microstructures and make it attractive enough to be considered for commercial applications. In this paper, a compact LED-based PµSL 3D printer for the fabrication of 3D microstructures was developed, and the effective parameters that influence the quality of construction were thoroughly investigated and optimized. Accordingly, a customized optical system, including illumination optics and projection optics, was designed using optical engineering principles. This custom 3D printer was proposed for the PµSL process, which besides improving the quality of construction, led to the reduction of the size of the device, its cost-effectiveness, and the repeatability of its performance. To demonstrate the performance of the fabricated device, a variety of complex 3D microstructures such as porous, hollow, helical, and self-support microstructures were constructed. In addition, the repeatability of the device was assessed by fabricating microstructure arrays. The device performance showed that the lateral accuracy of printing was better than 5 μm, and the smallest thickness of the printed layer was 1 μm. Moreover, the maximum printable size of the device was 6.4 mm × 4 mm × 40 mm.

## Introduction

In the past decade, the development of additive manufacturing or three-dimensional (3D) printing has grown rapidly due to its attractiveness and also the investments in research and technology^1^. There are several methods for 3D printing, such as fused deposition modeling^2^, inkjet printing^3^, stereolithography^4^, etc. that directly manufacture 3D models with a single process. For fabricating 3D microstructures, 3D printing techniques that are mainly based on photopolymerization reactions have emerged as an effective approach to print objects with micrometric or sub-micrometric resolutions. In fact, the main factors in choosing the construction process are accuracy, printing speed, material range, and dimensions of the desired object. According to the above considerations, the appropriate methods are microstereolithography (µSL)^5,6^, projection microstereolithography (PµSL)^7,8^, and two-photon polymerization (TPP)^9^.

In the μSL approach, a 3D microstructure is built using an ultraviolet (UV) laser scanning technique and a localized photopolymerization process. In this method, the 3D object is constructed additively, layer by layer, according to the pattern defined by scanning the laser beam. If in the illumination optic, the laser scanner is replaced by a spatial light modulator such as liquid crystal display (LCD)^10^, liquid crystal on silicon (LCOS)^11^, or digital micro-mirror device (DMD)^12^ as a dynamic mask generator, the method is called the PμSL. The main advantage of the PµSL over the µSL is its printing speed^13^. The PµSL 3D printer projects a 2D pattern of each layer into the photopolymer resin at the same time, and the cured layer is built with a single exposure. This means that the curing process in the PµSL 3D printer is much faster than point to point curing in a laser scanner.

Furthermore, this system has the fewest mechanical moving parts and requires only one accurate z-axis motorized linear stage. Therefore, in addition to guaranteeing the accuracy, the PµSL reduces the cost of construction and maintenance. Currently, a variety of materials are being used for the PµSL, including polymers, shape-memory polymers, ceramics, and biomaterials, which have increased its applications in various fields such as micro-optics^14^, metamaterials^15,16^, microfluidics^17–20^, tissue engineering^8,12,21–25^, and biomedical microdevices^26,27^.

To achieve further printing accuracy, two-photon polymerization has been introduced^9,28,29^. In this method, a pulsed femtosecond laser is used directly to create a 3D pattern in the depth of the photosensitive resin. In the TPP process, due to the nonlinear effects of the photosensitive resin at the tip of the focal point of the femtosecond laser, two infrared photons are absorbed, and a UV photon is generated which initiates the photopolymerization reaction. The 3D object is built by scanning the focal point inside the photosensitive resin in the three directions of *x*, *y*, and *z*. Although this method provides the highest accuracy among 3D printers, its applications are limited by the high cost of TPP components (such as femtosecond laser, scanning system, and optical elements), as well as the low printing speed, expensive consumable materials, and the small dimensions of the fabricated object^16^.

Therefore, the PµSL is a promising high-resolution 3D printing technique in terms of simplicity, accuracy, fast production time, cost-effectiveness, and the variety of materials that it uses^7^. However, it still requires further improvements to overcome its present limitations and to become attractive enough to be considered for commercial applications. In order to enhance the overall performance of the PµSL and increase its applications, many studies have focused on optical and hardware configuration^20,30–34^, resin formulation^35–39^, process planning optimization^40–43^, solidification modeling^37,44–46^, and multi-material printing^47–49^. In the mentioned studies, they used multi-purpose commercial optical components, and meanwhile, there were no reports on customizing optical components and reducing the device dimensions. The challenge with using the multi-purpose commercial optical components is that the size of the PμSL 3D printer and the accuracy of construction restricted by the characteristics of these components. Based on our knowledge, the development of the most compact PμSL 3D printer was reported by Gong *et al*.^20^. They developed a custom PμSL 3D printer with a lateral resolution of 7.6 μm to produce microfluidic flow channels. In this 3D printer, a commercial digital light processing engine (Visitech, Lier, Norway) consisting of illumination optics and projection optics was used.

In this study, we have aimed to design and develop a compact LED-based PμSL 3D printer for the fabrication of high-resolution 3D microstructures. The volume of our fabricated 3D printer, about 90% for the digital light processing engine, and 50% for the overall size of the device is smaller than the Gong group 3D printer. In this regard, a customized optical system was designed using optical engineering principles, and the effective parameters in the fabrication of 3D microstructures using projection microstereolithography (PµSL) technique were thoroughly investigated. In addition, conventional photopolymer resin and UV light absorber were used. Then, the resin formulation was optimized by a selection process based on a few tries of light absorber concentrations according to the light source properties. This method is also applicable to other photopolymer resins such as some hydrogels and so on. A DMD chip was used as a spatial light modulator to generate the dynamic mask of UV exposure.

In order to minimize the final dimensions of the 3D printer, all the parts were engineered and assembled into a compact size. In this study, the device controller and the slicer program were developed and implemented. By these two programs, the 3D printer has the ability to fabricate a 3D microstructure with different layer thicknesses and adjust the printing speed simultaneously according to the degree of complexity in different parts of the microstructure. To illustrate the potential of the proposed system, its performance has been demonstrated by printing various objects with 3D complexity such as porous or hollow microstructures, microstructures with 3D curvature, and 3D microstructures without mechanical support.

## Materials and Methods

### System configuration

The PµSL has two configurations depending on the orientation of the light source: top-down projection approach (free surface technique) and bottom-up orientation approach (constrained surface technique)^50^. In both configurations, the 3D microstructure is made additively in a layer-by-layer method, which starts from the bottom layer of the model to the top layer.

Compared with the top-down approach, the bottom-up approach can reduce the required amount of resin and is able to produce objects with high-viscosity materials. Moreover, in this configuration, the height of the printed object is not limited to the height of the resin tank, and the layer thickness can be accurately controlled^50^. The bottom-up configuration approach was chosen due to its advantages.

**Figure 1:**
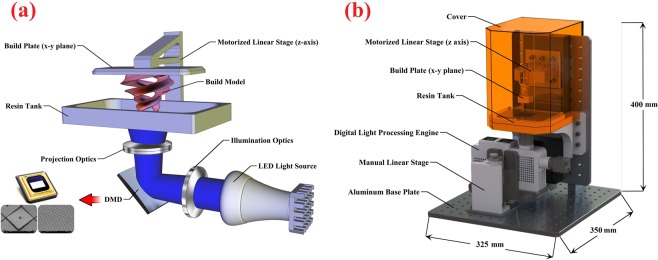
(**a**) The schematic illustration of the bottom-up configuration and (**b**) The 3D CAD model of the designed compact PµSL 3D printer (this figure was created by SOLIDWORKS 2016 (www.solidworks.com)).

The schematic illustration of the bottom-up configuration and the 3D CAD model of the designed compact PµSL 3D printer are depicted in Fig. 1.

According to Fig. 1, the fabricated 3D printer consists of a digital light processing engine, a resin tank, a build plate, and a motorized linear translation stage (Thorlabs, NRT100). The position of the digital light processing engine is under the resin tank and is adjusted by the manual linear translation stage to align the UV light projected pattern onto the transparent bottom of the resin tank. The bottom of the resin tank is a UV-transparent and non-stick Teflon film. The Teflon film is attached to a UV grade fused-silica window (with 1 mm thickness) as a rigid structure that can be held in the same vertical location for all layers. The dimensions of the resin tank were 30 mm × 25 mm × 15 mm. The build plate is also attached to the motorized linear translation stage and is located above the resin tank. The 3D object is printed hanging from the movable build plate. In this configuration, each newly cured layer is sandwiched between the previous layer and the resin tank.

To prevent the entry of dust and unwanted UV radiation into the resin tank during the printing process, a portable yellow plexiglass cover was placed on the build plate. To reduce the final size of the fabricated PµSL 3D printer, the dimensions of the digital light processing engine were optimized. Then all the optical components were assembled in a custom box that was designed for this purpose. The box model was drawn by SolidWorks software and produced using a commercial FDM 3D printer. Assembling all components in a compact box also improved the mobility and robustness of the system. The dimensions of the digital light processing engine were 17 cm × 4.5 cm × 12 cm. All the 3D printer components were mounted on an aluminum base plate. The overall dimensions of the fabricated PµSL 3D printer were 32.5 cm × 35 cm × 40 cm.

### Digital light processing engine

In the PµSL 3D printer, the digital light processing engine plays a vital role in printing 3D microstructures with a high resolution and quality. According to Fig. 1(a), the digital light processing engine of the PµSL 3D printer consists of three subsystems: illumination optics, DMD, and projection optics. The details of their design and fabrication are explained below.

#### Illumination optics

To ensure optimal illumination when designing a system, the role of each optical component must be properly recognized. In this study, the Zemax optical design software was used to design and optimize the illumination optics and to determine the specifications of the required optical components.

**Figure 2:**
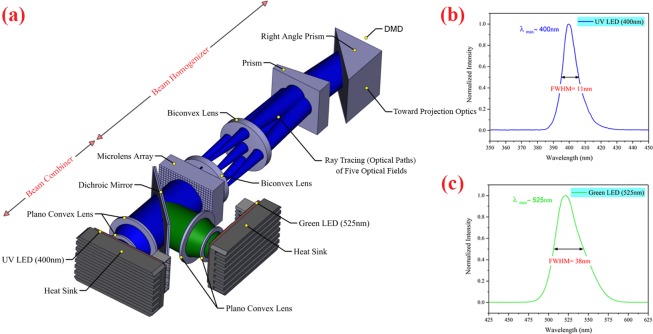
(**a**) The 3D CAD optical design of the illumination optics. In this design, the ray tracing (optical paths) of five optical fields are shown (Optical design was done by Zemax OpticStudio 2016 (www.zemax.com), and then 3D CAD optical model imported to SOLIDWORKS 2016 (www.solidworks.com) for creating the figure). (**b**) The relative spectrum of 400 nm UV LED (FWHM = 11 nm). (**c**) The relative spectrum of 525 nm green LED (FWHM = 38 nm).

In Fig. 2, the designed 3D CAD optical model of the illumination optics is shown. In this design, the illumination optics consist of two parts: the optical beam combiner and the optical beam homogenizer.

In the optical beam combiner section, two LED light sources were employed and collimated by two plano-convex lenses and then combined with a 450 nm short-pass dichroic mirror (Edmund Optics). The wavelengths of these LEDs were 400 nm (UV) and 525 nm (green). The former was considered for the photopolymerization process, while the latter was used for background illumination and monitoring of the curing process. The relative spectra of these LEDs were measured by a fiber-optic spectrometer (Avantes, AvaSpec-ULS2048L), as depicted in Fig. 2. The combined LED light sources were then directed into the optical beam homogenizer. The 450 nm short-pass dichroic mirror is highly transmissive (>90%) below the 450 nm wavelength and highly reflective (>97%) above that wavelength and is designed for a 45° angle of incidence. The optical power of the UV and green LEDs is tunable up to 4 watts and has a 2 mm × 2 mm illumination active area. It should be noted that the reason for using LED instead of a mercury lamp (which is mainly used in similar systems) is its advantages including its long lifetime (up to 30000 hours), energy efficiency, stable and highly tunable output power, uniform light output, fast response time, and small size.

**Figure 3:**
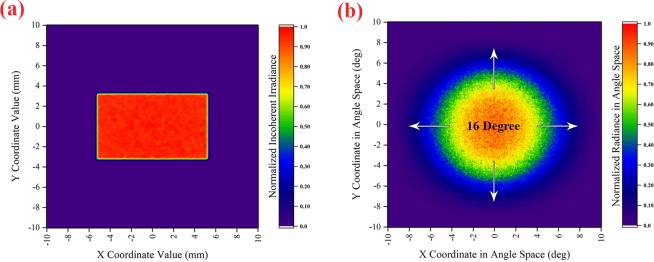
The simulation results of the (**a**) normalized incoherent Irradiance Distribution (**b**) angular distribution of normalized radiance of homogenized light on the surface of the DMD. The unit of angular distribution is degree and plotted respect to the normal vector of the DMD surface (this figure was created by Origin 2017 (www.originlab.com)).

Since the rate of curing and thickness of the cured layer depends on the radiation intensity, the uniformity of the layer thickness depends on the uniformity of the light intensity distribution. The optical beam homogenizer is another part of the illumination optics that collects the light from the optical beam combiner and then makes the intensity distribution of light uniform and directs it onto the DMD. To obtain maximum optical coupling efficiency from the illumination optics to the projection optics, the divergence angle of light from the homogenization plane (surface of the DMD) must be set equal to the numerical aperture (NA) of the projection optics. Also, the incident angle of light onto the surface of the DMD must be 24 degrees. The overall structure of the designed illumination optics and its components were optimized using the optical simulation method. The simulation results of the normalized incoherent irradiance distribution and angular distribution of the rays of the homogenized light on the surface of the DMD (divergence angle of light reflected by the DMD) are shown in Fig. 3.

According to the simulation results, the uniformity of irradiance distribution on the surface of the DMD is 94% (Fig. 3a). The uniformity of more than 90% in irradiation distribution gives the uniformity of more than 90% to the cured layer thickness. Therefore, the potential error caused by this non-uniformity of irradiation can be ignored in the manufacturing accuracy. In this study, the aim was that the divergence angle of light from the surface of the DMD for *1/e*^2^ of maximum radiance is 16 degrees (Fig. 3b). The obtained divergence angle was utilized in designing the projection optics, as shown in the simulation.

Based on the final design, the illumination optics were fabricated. To verify the results of the fabricated illumination optics, the uniformity of irradiance distribution on the entire surface of the DMD (where the homogenized light is produced) was measured by a lab-made motorized optical power meter using raster scan method and compared with the results of the optical simulation. This measurement device consists of an optical power meter (Thorlabs PM100) with a 5 µm pinhole added to the sensor head and an *x-y* motorized linear translation stage (Thorlabs PT3-Z8). In this experiment, an irradiance distribution uniformity of approximately 89% was measured on the surface of the DMD. The difference between the simulation and experimental results is due to the optical component performance, optical misalignment, and straylight effects.

#### DMD and projection optics

DMD is a spatial light modulator that uses micro-opto-electromechanical systems (MOEMS). A DMD consists of hundreds of thousands of movable micro-mirrors arranged in a 2D rectangular array. The mirrors can be individually rotated ±12° in an on or off state. In the on state, light from the illumination optics is reflected into the projection optics and produces a bright pixel on the image plane. In the off state, the light is directed into the beam dumper making the pixel appear dark^51^. In Fig. 1(a), the DMD is shown.

The DMD device used in this study (Texas Instruments, DLP4501 DMD module with the electronic board) has square-shaped micro-mirrors with a cross-section of 7.6 μm × 7.6 μm. The micro-mirrors are arranged in 2D arrays of 1280 × 800 pixels in a rectangular active area of 0.45 inches in diameter (9.7 mm × 6.1 mm).

In order to project UV radiation by DMD onto the surface of the photopolymer resin, it is necessary to design the projection optics. The accuracy of the lateral construction (construction in the *x-y* plane) of the printed objects depends on the optical resolution of the projection optics. In this design, the purpose is that the accuracy of printing reaches the range of microns, and the dimensions of the construction reach the range of millimeters.

In PμSL systems, there is a linear relationship between the lateral accuracy of printing and the maximum lateral dimensions of construction. The maximum lateral dimensions of construction is depends on the DMD active area and the magnification of the projection optics. Clearly, many magnifications could be designed for projection optics. In this system, according to the DMD active area and its number of pixels, designing two projection optics is attractive and functional. One of them for the lateral accuracy of printing in the range of 5 µm with the maximum lateral dimensions of 6.4 mm × 4 mm and the other for the lateral accuracy of printing in the range of 1 µm with the maximum lateral dimensions of 1.28 mm × 0.8 mm. In this paper, the lateral accuracy of printing in the range of 5 µm was selected, and the projection optics was designed accordingly.

**Figure 4:**
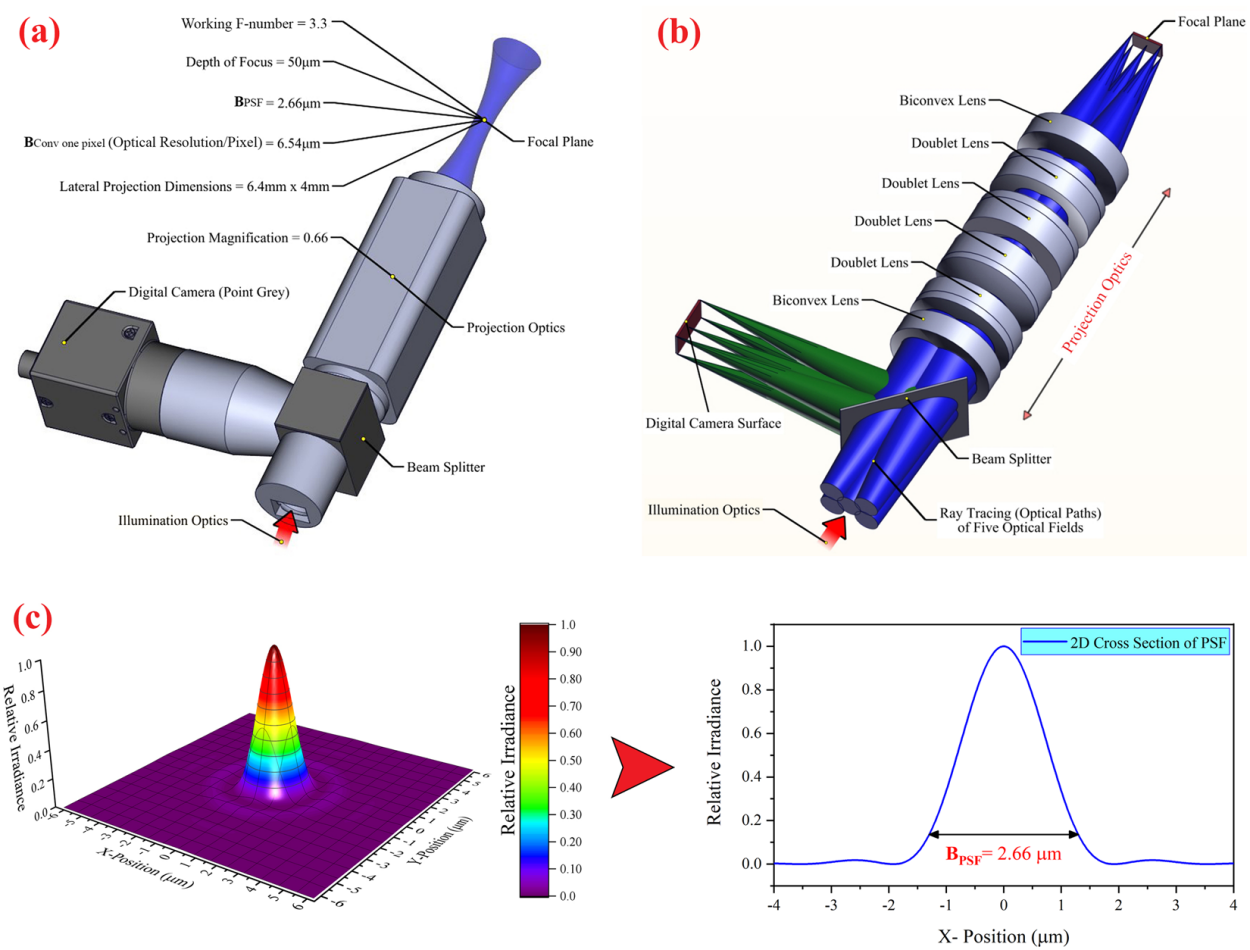
(**a**) The 3D CAD model of projection optics along with the optical parameters (this figure was created by SOLIDWORKS 2016 (www.solidworks.com)). (**b**) The 3D CAD optical design of the projection optics. In this design, the ray tracing (optical paths) of five optical fields are shown (Optical design was done by Zemax OpticStudio 2016 (www.zemax.com), and then 3D CAD optical model imported to SOLIDWORKS for creating the figure). (**c**) The PSF function and the 2D cross-section of PSF for the designed projection optics (this figure was created by Origin 2017 (www.originlab.com)).

In Fig. 4(a), the 3D CAD model of the projection optics is shown. In this design, a digital camera (Point Grey, Blackfly) and an optical beam splitter are used in the optical path for observing the image plane of projection optics and monitoring the curing process. In Fig. 4(b), the 3D CAD optical design of the projection optics and the optical paths of five optical fields are shown. According to Fig. 4(b), the projection optics consist of 10 lenses (four different doublet lenses and two different biconvex lenses) that optimized for the wavelength range of 350 to 600 nm.

In designing the projection optics, considering the effect of the dimensions of the micro-mirror of DMD on the width of the focal intensity distribution at the projection plane (optical resolution/pixel), the width of the optical point spread function^52^ (PSF) of the designed projection optics was about 2.6 µm for the wavelength of 400 nm. Selecting the width of 2.6 µm for the PSF was based on considering the lateral accuracy of about 5 µm. The width of the PSF (B_PSF_) is defined as the spot diameter at the *1/e*^2^ of maximum intensity.

In addition, due to the dimensions of the DMD active area, to achieve the maximum lateral dimensions of construction (6.4 mm × 4 mm), the magnification of the projection optics was designed with a value of 0.66. Also, the working F-number (the ratio of the system’s focal length to the diameter of the entrance pupil) of the projection optics was designed as 3.3 so that its depth of focus reached 50 μm. Accordingly, the effect of increasing the width of the PSF along the beam propagation (*z*-axis) can be ignored, and the condition of *ω*_0_*(z)* = *ω*_0_ is established in the following equations in section “numerical model.” In order to achieve the highest energy transfer efficiency between the illumination optics and projection optics, the NA of the projection optics was designed based on the divergence angle of light reflected by the DMD (Fig. 3(b)). In Fig. 4(c), the PSF function, and the 2D cross-section of the PSF for the designed projection optics are shown. As shown in Fig. 4(c), the B_PSF_ is equal to 2.66 µm.

### Numerical model

The spatial resolution of construction (the printing resolution) is the main issue in designing the PμSL 3D printer. Three factors that determine the spatial resolution of construction are optical resolution of the projection optics, exposure energy per unit area, and the physical-chemical characteristics of the photopolymer resin. As is clear, the optical resolution of projection optics is determined by PSF^52^. Due to the incoherency of the light reflected from DMD micro-mirrors and the linear response of the optical systems, the effect of micro-mirror dimensions on the final spread energy at the projection plane (optical resolution/pixel) is equal to the superposition of PSF of all the points on the micro-mirror surface. This superposition is calculated by the spatial convolution equation^53^. Equation 1 is the integral form of the spatial convolution equation:

1 $$f(x,y)\ast PSF(x,y)={\int }_{{\tau }_{1}=-\infty }^{\infty }{\int }_{{\tau }_{2}=-\infty }^{\infty }f({\tau }_{1},{\tau }_{2}).PSF(x-{\tau }_{1},y-{\tau }_{2})d{\tau }_{1}d{\tau }_{2}$$

where *f(x, y)* is the spatial function of the micro-mirror cross-section at the projection plane. *f(x, y)* for one pixel is obtained by Eq. 2:

2 $$f(x,y)=\{\begin{array}{l}1\,-\,m\frac{{d}_{x}}{2} < x < m\frac{{d}_{x}}{2}\,and-m\frac{{d}_{y}}{2} < y < m\frac{{d}_{y}}{2}\\ 0\,x < -\,m\frac{{d}_{x}}{2}\,and\,m\frac{{d}_{x}}{2} < x\,and\,y < -\,m\frac{{d}_{y}}{2}\,and\,m\frac{{d}_{y}}{2} < y\end{array}$$

where *d*_*x*_ and *d*_*y*_ are the sizes of the micro-mirror along the *x* and *y* axes, respectively, and *m* is the magnification of the projection optics.

**Figure 5:**
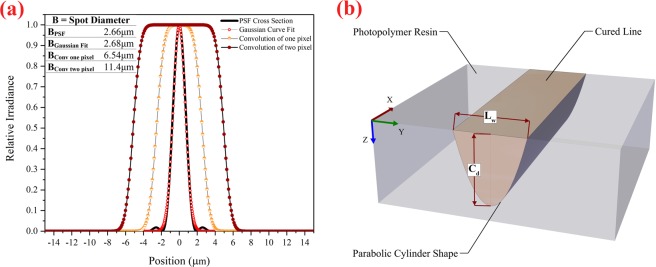
(**a**) The cross-section of the PSF function with its fitted Gaussian curve and the effect of the spatial convolution of 1 and 2 pixels on the width of the PSF function at the projection plane. (**b**) The schematic illustration of the cured line with a parabolic cylinder shape and two main photopolymerization parameters for determining the spatial resolution of construction in the PμSL technique (this figure was created by SOLIDWORKS 2016 (www.solidworks.com)).

To better understand the photopolymerization process in a PµSL 3D printer, the numerical model of a stereolithography 3D printer^54^ was modified. For this purpose, the Gaussian distribution function that is the first-order approximation of the PSF function was used^55^. Then, with the help of the spatial convolution equation (Eq. 1), the effect of DMD pixels on the radius of the equivalent Gaussian distribution function (*ω*_0_) in the focal plane of the projection optics was obtained and used in the following equations. In Fig. 5(a), the 2D cross-section of the PSF function with its fitted Gaussian curve and the effect of the spatial convolution of 1 and 2 pixels on the width of the PSF function are shown. In this figure, the optical resolution of the designed projection optics for the ideal point source (B_PSF_), for one pixel (B_Conv one pixel_), and for two pixels (B_Conv two-pixel_) has been calculated and shown.

The Gaussian intensity distribution of an ideal point source on the projection plane at the given positions of *x* and *y* is defined by Eq. 3^52^:

3 $$\begin{array}{rcl}I(x,y) & = & {I}_{max}{e}^{(\frac{-2({x}^{2}+{y}^{2})}{{{\omega }_{0}}^{2}})}\\ {I}_{max} & = & \frac{2P}{\pi {{\omega }_{0}}^{2}}\end{array}$$

where *I(j.cm*^*−2*^*.s*^*−1*^) is the intensity distribution of UV light, *P(j.s*^*−1*^) is the total power of UV light, and *ω*_0_ (Gaussian radius) is half-width at *1/e*^2^ of Gaussian maximum intensity (*I*_*max*_). The *x-y* plane is the focal plane of the projection optics and is located on the surface of the photopolymer resin. In addition, the exposure energy per unit area is defined by Eq. 4. In this equation, *t(s)* is the exposure time (in seconds).

4 $$E(x,y,t)=I(x,y)t$$

When UV radiation enters the photopolymer resin, it is absorbed or scattered by the resin. These two effects determine the penetration depth of the resin and polymerization thickness. If the effect of scattering is ignored, the distribution of energy per unit area inside the resin surface can be determined by means of the Lambert-Beer absorption law. The energy distribution per unit area inside the resin surface that is reflected from each pixel of the DMD is defined by Eq. 5:

5 $$\begin{array}{rcl}E(x,y,z,t) & = & E(x,y,t){e}^{(-\frac{z}{{D}_{p}})}\\ {D}_{p} & = & \frac{1}{{\varepsilon }_{d}[D]+{\varepsilon }_{i}[S]\,}\end{array}$$

where *D*_*p*_ is the penetration depth of the resin. *D*_*p*_ is also referred to as a depth inside the resin in which the intensity of UV radiation is equal to *1/e* of the intensity on the surface of the resin. *ε*_*d*_ and *ε*_*i*_ are molar absorption coefficients of the photoinitiator and the light absorber, respectively. *D* and *S* are the concentrations of the photoinitiator and light absorber of the photopolymer resin, respectively. In this equation, it is assumed that the UV light enters the resin at *z* = 0 (resin surface) and propagates in it in the +*z* direction.

If a linear array of the DMD pixels with equal exposure energies is active along the *x-*axis, the energy distribution per unit area generated by projection optics inside the resin will be calculated by the following equation:

6 $$\begin{array}{c}E(y,z,t)=I(y,z)t=I(y)t{e}^{(-\frac{z}{{D}_{p}})}={I}_{max}{e}^{(\frac{-2{y}^{2}}{{{\omega }_{0}}^{2}})}t{e}^{(-\frac{z}{{D}_{p}})}=\frac{2Pt}{\pi {{\omega }_{0}}^{2}}{e}^{(\frac{-2{y}^{2}}{{{\omega }_{0}}^{2}}-\frac{z}{{D}_{p}})}\\ {E}_{max}={I}_{max}t=\frac{2Pt}{\pi {{\omega }_{0}}^{2}}\end{array}$$

This energy distribution produces a cured line with a parabolic cylinder shape, as depicted in Fig. 5(b). As is observed in Fig. 5(b), the maximum cured depth (*C*_*d*_) and maximum cured line width (*L*_*w*_) are two main photopolymerization parameters for determining the spatial resolution of construction in the PμSL technique, as defined in Eqs. 7 and 8.

7 $${C}_{d}={D}_{p}\,\mathrm{ln}(\frac{{E}_{max}}{{E}_{c}}),{C}_{d}={D}_{p}\,\mathrm{ln}(\frac{t}{{T}_{c}})$$

8 $${L}_{w(Gaussianfit)}={B}_{(Gaussianfit)}\sqrt{\frac{{C}_{d}}{2{D}_{p}}}$$

where *Ε*_*c*_ is the critical exposure energy, and *T*_*c*_ is the critical exposure time. *Ε*_*c*_ and *T*_*c*_ are the threshold energy and threshold time needed to start the solidification process. In fact, the photopolymerization process begins immediately upon illumination, but when the UV radiation of the resin surface exceeds the amount of *E*_*c*_ (*E*_*max*_ > *E*_*c*_), the resin reaches a point in the gelation process that it can be considered to be a solid (or nearly solid). Otherwise, the resin will remain liquid. The parameter *Β*_*Gaussian fit*_ = 2*ω*_*Gaussian fit*_ is the spot diameter of the Gaussian beam (full width at the *1/e*^2^ of maximum intensity) on the resin surface. Due to the linear effect of spatial convolution on the optical resolution of DMD pixel and according to Fig. 5(a), the produced maximum cured line width of the N-pixel-wide line (L_w(Conv N pixel)_) is obtained by Eq. 9.

9 $${L}_{w(ConvNpixel)}=({B}_{(ConvNpixel)}-{B}_{(Gaussianfit)})+{B}_{(Gaussianfit)}\sqrt{\frac{{C}_{d}}{2{D}_{p}}}$$

The two parameters *D*_*p*_ and *E*_*c*,_ are related to the physical-chemical properties of resin and are optimized by resin formulations. However, the UV exposure energy is controlled by the electronic part of the device with a high precision. As a result, the maximum depth of curing (thickness of the cured layer or vertical accuracy along the *z*-axis) and the maximum width of curing (lateral accuracy on the *x-y* plane) are controllable during the printing process. Since exposure energy per unit area (*E* = *It*) is a function of exposure intensity per unit area and exposure time, it can be controlled by exposure intensity or exposure time or both.

### Resin formulation and surface treatment

#### Chemicals

1,6-Hexanediol diacrylate (HDDA), phenylbis(2,4,6-trimethylbenzoyl) phosphine oxide (Irgacure 819), 1-phenylazo-2-naphthol (Sudan I), and 3-(trimethoxysilyl) propyl methacrylate (TMSPM) were purchased from Sigma Aldrich. NaOH, methanol, and isopropanol were obtained from Merck.

#### Resin formulation

The resin used in this work is composed of an acrylate-based commercial monomer (HDDA), a photoinitiator (Irgacure 819), and Sudan I (an absorber which adjusts the light penetration depth). The resins were prepared by mixing 2.6% (w/w) Irgacure 819 in HDDA along with variable amounts of Sudan I (0.1%, 0.25%, 0.5% w/w). The mixtures were sonicated in the dark for 40 min at 40 °C. Then, the stock solutions were kept in the dark place before being used. To remove the non-polymerized resin after printing, the printed objects were washed with isopropanol and dried under a stream of nitrogen.

#### Surface treatment

In order to increase the adhesion between the constructed microstructures and the glass surface, the slides were silanized using TMSPM before printing^56,57^. For this purpose, the slides were sonicated in NaOH (1.0 M) for 10 min, washed with distilled water, and dried under a stream of nitrogen. Then the activated slides were immersed in TMSPM-methanol solution (10/90, v/v) for 30 min at 50 °C. Finally, the silanized glasses were washed with isopropanol and dried under vacuum. The effect of surface treatment is shown in electronic supplementary information.

### 3D printer characterization

To verify the printing performance of the PμSL system, the printing resolution of the 3D objects was evaluated. The printing resolution consists of the accuracy of fabrication in the *x-y* plane (lateral accuracy) and in the *z-*direction (vertical accuracy).

#### Vertical accuracy characterization (in the *z-*axis direction)

Based on Eq. 7, the thickness of the cured layer or vertical accuracy of printing is directly related to the natural logarithm of the exposure energy per unit area. According to Eq. 5, since the molar absorption coefficient of Sudan I at the wavelength of 400 nm is high, the concentration of this substance in the resin formulation is more effective on the light penetration depth and consequently on the cured layer thickness compared to those of other materials. In fact, to optimize the printing resolution, it is necessary to adjust the concentration of Sudan I based on the UV exposure energy. In order to obtain a precise relationship between the concentration of Sudan I and the time required to print the desired layer thickness, the following procedure was adopted. A rectangular-shaped PMMA ring spacer with a thickness of 500 μm was placed between the silanized and the untreated slides, and then the empty space was filled by resin with different concentrations of Sudan I. Then, it was placed on the printing location. At the radiant intensity of *7.1 mW/cm*^2^, 10 rectangular strips were printed on the silanized slide with the exposure time of 350 to 2150 milliseconds at the intervals of 200 milliseconds. Afterward, to remove the non-polymerized resin, the slide was washed with isopropanol and dried under a stream of nitrogen. These steps were performed for three mixtures of 0.1, 0.25, and 0.5% (w/w) of Sudan I in the resin formulation and were repeated three times for each concentration. Finally, a Dektak XT profilometer (Bruker Corporation) was used to evaluate the thickness of the printed layers.

#### Lateral accuracy characterization (in the *x-y* plane)

In this section, similar to the previous section, a rectangular-shaped PMMA ring spacer with a thickness of 500 μm was placed between the silanized and the untreated slides, and then the empty space was filled by resin with 0.25% concentration of Sudan I. Then, in order to characterize the lateral accuracy of printing at the radiant intensity of *7.1 mW/cm*^2^, 20 lines with a width of one pixel and a distance of two pixels from each other were printed on the silanized slide for three exposure times at 790, 970, and 1580 milliseconds. To remove the non-polymerized resin, the slide was washed with isopropanol and dried under a stream of nitrogen. Finally, a Dektak XT (Bruker Corporation) profilometer was used to evaluate the 2D cross-section of the printed lines. The reason for choosing the resin with the concentration of 0.25% Sudan I and the selected exposure times for the test were the results of the thickness measurement of the layer in section “vertical accuracy characterization”.

### Software design

The software used for the 3D printer included the slicer and controller programs and was developed in the LabVIEW programming language environment. In the slicer program, at first, a 3D CAD file (STL file format) is converted into 2D layers in scalable vector graphics (SVG) file format. Using this format makes it possible to scale the size of 2D layers if needed without any loss of quality. In addition, the final file size of 2D layers is reduced. This feature is useful when printing long-length microstructures with 1 µm layer thickness due to the large number of 2D layers produced. The second feature of the slicer program is the generation of 2D layers with different thicknesses in one print. In this case, depending on the complexity and precision of the 3D microstructure, it is possible to change the thickness of various segments and optimize the print speed.

In the 3D printer controller program, one can set all the required parameters such as exposure intensity, exposure time, printing resolution, printing speed, controlling the printing process by a vision camera, and so on. Also, by entering the calibration function inside the program, the thickness of the layer is determined by the exposure intensity and exposure time associated with each resin formulation, as obtained in section “vertical accuracy characterization”. Therefore, the controller program controls the print process for different thicknesses from the beginning to the end.

### 3D microstructure fabrication

To demonstrate the capability of the presented PµSL system, some objects with various 3D complex microstructures were generated. The fabrication characteristics of the printed objects were investigated using scanning electron microscopy (SEM, HITACHI S-4160).

## Results and Discussion

### Vertical accuracy characterization

In order to demonstrate the role of the effective parameters on vertical accuracy, several rectangular strips were printed on the glass surface under different exposure times and light absorber concentrations, and then the thickness of each strip was measured.

**Figure 6:**
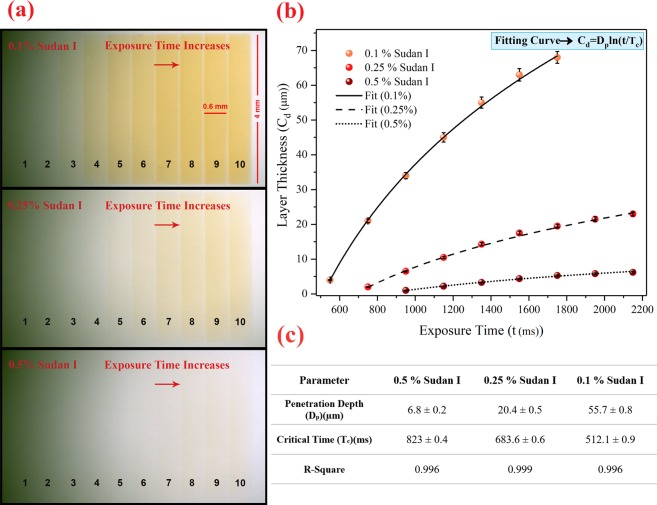
Characterization of the vertical accuracy of printing. (**a**) Microscopic image of rectangular strips printed in a single layer with exposure time in the range of 350 to 2150 milliseconds with a time interval of 200 milliseconds for three types of resins with different concentrations of Sudan I (0.1%, 0.25%, 0.5% w/w), (**b**) The cured layer thickness graph versus the exposure time and concentration of Sudan I (experimental results), (**c**) The penetration depth and critical time for three concentrations of Sudan I obtained from the fitting curve of the experimental results.

Figure 6(a) shows a microscopic image of rectangular stripes printed in a single layer with an exposure time in the range of 350 to 2150 milliseconds with a time interval of 200 milliseconds for three types of resins with different concentrations of Sudan I (0.1%, 0.25%, 0.5% w/w). In Fig. 6(b), the results of measuring the thickness of the cured rectangular stripes (*C*_*d*_) are shown versus the exposure times (*t*) for the three concentrations of Sudan I in the resin formulations. These results are fitted by Eq. 7, and two parameters penetration depth (*D*_*p*_) and critical time (*T*_*c*_) (relating to the physical-chemical properties of the resin) for the three concentrations of Sudan I was calculated. These parameters are shown in table 6(c). In this test, the UV exposure intensity was set to *7.1 mW/cm*^*2*^. According to Fig. 6(b), for the resin with 0.5% Sudan I, the layer thickness can be controlled in the range of 500 nm to 6 μm by the UV exposure time (in the range of 900 to 2200 ms). In addition, for the resin with 0.25% Sudan I, the UV exposure time in the range of 700 to 2200 ms can control the layer thickness in the range of 1 to 22 μm. Also, for the resin with 0.1% Sudan I, the layer thickness can be controlled in the range of 2 to 70 μm by the UV exposure time in the range of 500 ms to 1800 ms.

Based on the repeatability test of the thickness of the layers, by increasing the concentration of Sudan I, the accuracy of controlling the layer thickness is increased.

### Lateral accuracy characterization

**Figure 7:**
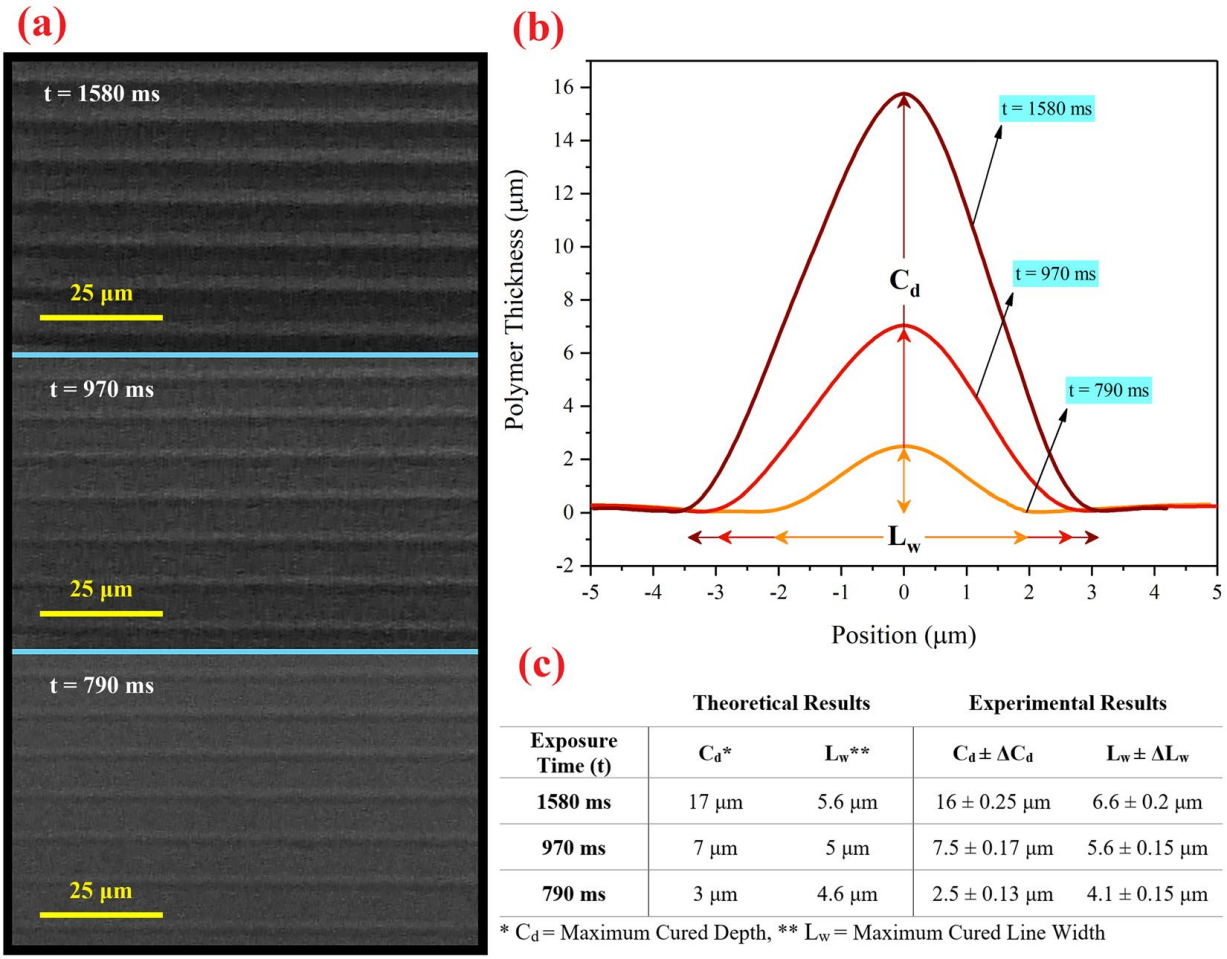
Characterization of the lateral accuracy of printing. (**a**) The SEM images of the printed lines, (**b**) Cross-section graph of one printed line for the indicated times. (**c**) The table showing the experimental and theoretical results.

Based on the results of Fig. 6, the resin with the concentration of 0.25% Sudan I, which provides control over the layer thickness in the range of 1 to 22 μm, was selected. This resin was used to check the lateral accuracy of printing. For this purpose and test the results, several 1-pixel-wide lines with the heights of (*C*_*d*_) 17, 7, and 3 μm were randomly chosen and printed on the surface of the glass. To obtain the polymerization parameters, the exposure time and the maximum cured line width were calculated using the results of Eq. 7, Eq. 9 (N = 1 for 1-pixel-wide line), and the table in Fig. 6(c). These results are presented in Fig. 7(c) in the theory section. In order to characterize the lateral accuracy of printing and compare it with theoretical results, 20 lines with the width of one pixel and the distance of two pixels from each other were printed for three exposure times (at 790, 970, and 1580 milliseconds). Then the cross-sections of the printed lines were measured by the DektakXT profilometer. In Fig. 7(a), the scanning electron microscope images of the printed lines in three exposure times of 790, 970, and 1580 milliseconds are shown. Figure 7(b) shows the measured cross-section of the printed line. The results of the measurement are presented in Fig. 7(c) and the experimental and theoretical results are in good agreement.

### 3D microstructure fabrication

**Figure 8:**
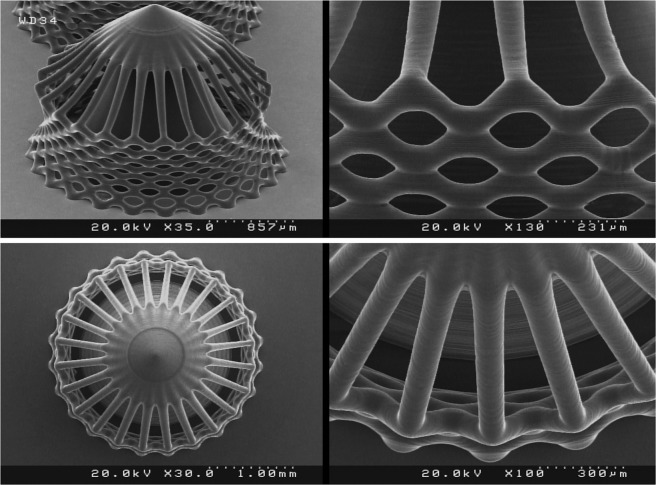
The performance of the system in the construction of complicated hyperboloid microstructures with thin and tall 2D lattices without the use of any support. The smallest diameter of the lattice structures is about 50 μm. The smallest linear hole dimension is about 40 μm. The smallest diameter of the cylinder’s rods is 80 μm, and its length is 700 μm. The build angle of the cylinders is 45 degrees relative to the horizon. The 3D microstructure is made with a diameter of about 2.66 mm and a height of about 2.2 mm. The thickness of the layers is set to 2 μm.

**Figure 9:**
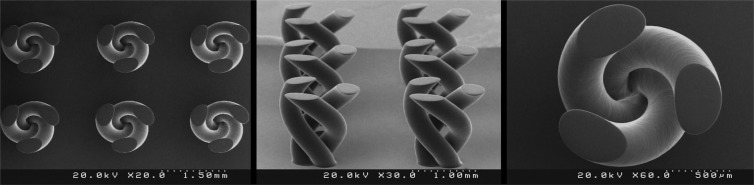
The performance of the system in the construction of microstructures with 3D curvature in an arrayed manner (helical microstructure). The thickness of the layers is set to 1 μm. The height of the microstructure is 1.3 mm.

**Figure 10:**
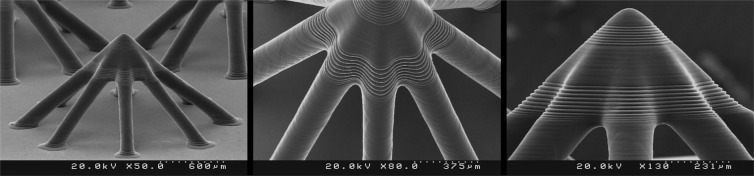
The performance of the system in the multi-thickness construction of one object and the growth of tall, angled, and thin cylindrical rods without any support. An oval cross-sectional cylindrical structure with an approximate diameter of 100 μm and a length of about 850 μm at a build angle of about 45° relative to the horizon. The thicknesses of layers are set to 1, 5, and 10 μm randomly at different parts of the microstructure. The height of the microstructure is 1.1 mm.

**Figure 11:**
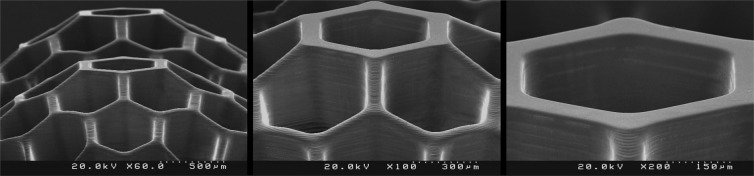
The performance of the system in the construction of microstructures with tall and thin walls. The minimum width of the walls in the honeycomb network is about 40 μm. The thickness of the layers is set to 3 μm. The height of the microstructure is 2.1 mm.

**Figure 12:**
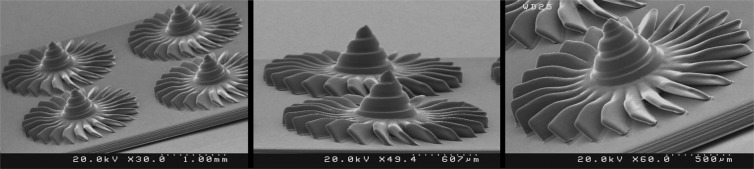
Micro-fan. The performance of the system in printing a complex microstructure object in which plates with a build angle of about 45° relative to the horizon and a thickness of 35 μm have been grown. The thickness of the layers is set to 1 μm.

**Figure 13:**
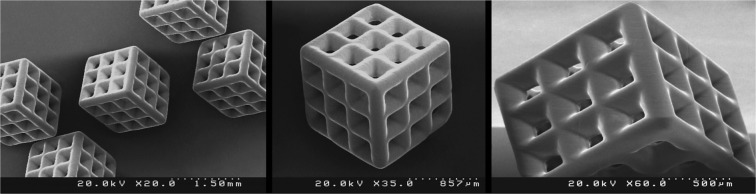
The performance of the system in printing a network of cross-microchannels. As is clear, microchannels are angled relative to the horizon. The dimensions of the printed cubes are 1.25 mm × 1.25 mm × 1.25 mm. The cross-sections of the microchannels are 100 μm × 100 μm.

In order to evaluate the performance of the fabricated PµSL printer, some microstructure models were generated in millimeter dimensions and micrometer resolutions. The selected models have various complex 3D microfeatures such as porous, hollow, helical, and self-supported microstructures. Figures 8 to 13 show the SEM micrographs of these printed objects. In Fig. 8, a complicated hyperboloid microstructure with a lattice wall is shown. In this object, the long, narrow, and angled rods were printed without any support. In Fig. 9, the cured depth was well adjusted to satisfy the vertical resolution down to 1 μm. This feature is very important for the fabrication of the curved parts. In addition, the reproducibility was demonstrated by printing the objects in an arrayed manner. A structure with multi-thickness layers was printed, as is shown in Fig. 10. The conical part of this structure was printed with 1, 5, and 10 μm layer thicknesses and constructed on narrow rods with a 45° angle relative to the horizon. In Fig. 11, the honeycomb network with tall and thin walls is shown. In Fig. 12, micro-fans printed in an arrayed manner are shown. In Fig. 13, an array of the printed cubes with the dimensions of 1.25 mm × 1.25 mm × 1.25 mm is shown. These cubes have been grown on one of their corners. Within these cubes, a network of cross-microchannels with a cross-section of 100 μm × 100 μm has been constructed.

## Conclusion

The PµSL technique shows promise as a future additive manufacturing technique for the construction of various complex 3D microstructures owing to its *x-y-z* resolution, facile application, and printing speed. In this study, a compact LED-based PµSL 3D printer that can fabricate mesoscale structures with micro-scale features was developed. For this purpose, the optical system, which consists of illumination optics and projection optics, was designed and developed based on optical engineering principles. To better understand the photopolymerization process in a PµSL 3D printer, the numerical model of the stereolithography 3D printer was modified, and the parameters that affect the quality of construction were investigated. The device performance showed that the lateral accuracy of printing was better than 5 μm and its minimum vertical accuracy was 1 μm. The controller and slicer programs were developed and implemented. By these two programs, the 3D printer has the ability to fabricate a 3D microstructure with different layer thicknesses and adjust the printing speed simultaneously according to the degree of complexity in different parts of the microstructure. Finally, by using the developed PμSL system, a variety of complicated 3D microstructures with a high resolution and reproducibility were fabricated. It is expected that the development of this 3D printer will benefit many researchers by fabricating 3D microstructures with complex geometries and reducing the costs.

## Supplementary information

Supplemental Information.

## References

[1] Ngo TD, Kashani A, Imbalzano G, Nguyen KTQ, Hui D (2018). Additive manufacturing (3D printing): A review of materials, methods, applications and challenges. Compos. Part B Eng..

[2] Mohamed OA, Masood SH, Bhowmik JL (2015). Optimization of fused deposition modeling process parameters: a review of current research and future prospects. Adv. Manuf..

[3] Shirazi SFS (2015). A review on powder-based additive manufacturing for tissue engineering: selective laser sintering and inkjet 3D printing. Sci. Technol. Adv. Mater..

[4] Melchels FPW, Feijen J, Grijpma DW (2010). A review on stereolithography and its applications in biomedical engineering. Biomaterials.

[5] Accardo A, Courson R, Riesco R, Raimbault V, Malaquin L (2018). Direct laser fabrication of meso-scale 2D and 3D architectures with micrometric feature resolution. Addit. Manuf..

[6] You, S., Miller, K. & Chen, S. Microstereolithography. In *Biofabrication and 3D Tissue Modeling* 1–21 (2019).

[7] Choi, J.-W., Lu, Y. & Wicker, R. B. Projection Microstereolithography as a Micro-Additive Manufacturing Technology: Processes, Materials, and Applications. in *Additive Manufacturing* 114–142 (CRC Press, 2015).

[8] Choi JW (2009). Fabrication of 3D biocompatible/biodegradable micro-scaffolds using dynamic mask projection microstereolithography. J. Mater. Process. Technol..

[9] Lin Y, Xu J (2018). Microstructures Fabricated by Two-Photon Polymerization and Their Remote Manipulation Techniques: Toward 3D Printing of Micromachines. Adv. Opt. Mater..

[10] Wang Z, Martin N, Hini D, Mills B, Kim K (2017). Rapid Fabrication of Multilayer Microfluidic Devices Using the Liquid Crystal Display-Based Stereolithography 3D Printing System. 3D Print. Addit. Manuf..

[11] Lazarev, G., Hermerschmidt, A., Krüger, S. & Osten, S. LCOS spatial light modulators: Trends and Applications. *Opt. Imaging Metrol. Adv. Technol*. 1–29, 10.1002/9783527648443.ch1 (2012).

[12] Lu Y, Mapili G, Suhali G, Chen S, Roy K (2006). A digital micro-mirror device-based system for the microfabrication of complex, spatially patterned tissue engineering scaffolds. J. Biomed. Mater. Res. Part A.

[13] Vaezi M, Seitz H, Yang S (2013). A review on 3D micro-additive manufacturing technologies. Int. J. Adv. Manuf. Technol..

[14] Camposeo, A., Persano, L., Farsari, M. & Pisignano, D. Additive Manufacturing: Applications and Directions in Photonics and Optoelectronics. *Adv. Opt. Mater*. **1800419** (2018). 10.1002/adom.201800419 PMC6358045 30775219

[15] Chen D, Zheng X (2018). Multi-material Additive Manufacturing of Metamaterials with Giant, Tailorable Negative Poisson’s Ratios. Sci. Rep..

[16] Mao M (2017). The Emerging Frontiers and Applications of High-Resolution 3D Printing. Micromachines.

[17] Gong H, Woolley AT, Nordin GP (2018). 3D printed high density, reversible, chip-to-chip microfluidic interconnects. Lab Chip.

[18] Waheed S (2016). 3D printed microfluidic devices: enablers and barriers. Lab Chip.

[19] Bhattacharjee N, Urrios A, Kang S, Folch A (2016). The upcoming 3D-printing revolution in microfluidics. Lab Chip.

[20] Gong H, Bickham BP, Woolley AT, Nordin GP (2017). Custom 3D printer and resin for 18 μm × 20 μm microfluidic flow channels. Lab Chip.

[21] Zhang R, Larsen NB (2017). Stereolithographic hydrogel printing of 3D culture chips with biofunctionalized complex 3D perfusion networks. Lab Chip.

[22] Gauvin R (2012). Microfabrication of complex porous tissue engineering scaffolds using 3D projection stereolithography. Biomaterials.

[23] Han L-H, Mapili G, Chen S, Roy K (2008). Projection Microfabrication of Three-Dimensional Scaffolds for Tissue Engineering. J. Manuf. Sci. Eng..

[24] Ronca A, Ambrosio L (2017). Polymer based scaffolds for tissue regeneration by stereolithography. Adv. Biomater. Devices Med..

[25] Chartrain NA, Williams CB, Whittington AR (2018). A review on fabricating tissue scaffolds using vat photopolymerization. Acta Biomater..

[26] Scholten K, Meng E (2015). Materials for microfabricated implantable devices: A review. Lab Chip.

[27] Yun H, Kim H (2013). Development of DMD-based micro-stereolithography apparatus for biodegradable multi-material micro-needle fabrication. J. Mech. Sci. Technol..

[28] Lv C (2018). Humidity-responsive actuation of programmable hydrogel microstructures based on 3D printing. Sensors Actuators, B Chem..

[29] Sun YL (2012). Dynamically tunable protein microlenses. Angew. Chemie - Int. Ed..

[30] Sun C, Fang N, Wu DM, Zhang X (2005). Projection micro-stereolithography using digital micro-mirror dynamic mask. Sensors Actuators, A Phys..

[31] Cooper GJT (2015). Development of a 3D printer using scanning projection stereolithography. Sci. Rep..

[32] Zheng X (2012). Design and optimization of a light-emitting diode projection micro-stereolithography three-dimensional manufacturing system. Rev. Sci. Instrum..

[33] Choi JW, Ha YM, Lee SH, Choi KH (2006). Design of microstereolithography system based on dynamic image projection for fabrication of three-dimensional microstructures. J. Mech. Sci. Technol..

[34] Ha YM, Choi JW, Lee SH (2008). Mass production of 3-D microstructures using projection microstereolithography. J. Mech. Sci. Technol..

[35] Simfukwe J, Mapasha RE, Braun A, Diale M (2017). Biopatterning of Keratinocytes in Aqueous Two-Phase Systems as a Potential Tool for Skin. Tissue Engineering. MRS Adv..

[36] Bennett J (2017). Measuring UV curing parameters of commercial photopolymers used in additive manufacturing. Addit. Manuf..

[37] Gong H, Beauchamp M, Perry S, Woolley AT, Nordin GP (2015). Optical approach to resin formulation for 3D printed microfluidics. RSC Adv..

[38] Kowsari K (2018). Photopolymer formulation to minimize feature size, surface roughness, and stair-stepping in digital light processing-based three-dimensional printing. Addit. Manuf..

[39] Qaderi, K. Polyethylene Glycol Diacrylate (PEGDA) Resin Development for 3D-Printed Microfluidic Devices. *Brigham Young Univ. Theses* (2015).

[40] Limaye AS, Rosen DW (2007). Process planning method for mask projection micro‐stereolithography. Rapid Prototyp. J..

[41] Fang N, Sun C, Zhang X (2004). Diffusion-limited photopolymerization in scanning micro-stereolithography. Appl. Phys. A.

[42] Park IB, Ha YM, Lee SH (2011). Dithering method for improving the surface quality of a microstructure in projection microstereolithography. Int. J. Adv. Manuf. Technol..

[43] Männel MJ, Selzer L, Bernhardt R, Thiele J (2018). Optimizing Process Parameters in Commercial Micro-Stereolithography for Forming Emulsions and Polymer Microparticles in Nonplanar Microfluidic Devices. Adv. Mater. Technol..

[44] Wu J, Lee NY (2014). One-step surface modification for irreversible bonding of various plastics with a poly(dimethylsiloxane) elastomer at room temperature. Lab Chip.

[45] Kang H-W, Park JH, Cho D-W (2012). A pixel based solidification model for projection based stereolithography technology. Sensors Actuators A Phys..

[46] Emami MM, Barazandeh F, Yaghmaie F (2015). An analytical model for scanning-projection based stereolithography. J. Mater. Process. Technol..

[47] Pan J, Tu S, Wang C, Chang J (2008). High efficiency pocket-size projector with a compact projection lens and a light emitting diode-based light source system. Appl. Opt..

[48] Kowsari K, Akbari S, Wang D, Fang NX, Ge Q (2018). High-Efficiency High-Resolution Multimaterial Fabrication for Digital Light Processing-Based Three-Dimensional Printing. 3D Print. Addit. Manuf..

[49] Choi JW, MacDonald E, Wicker R (2010). Multi-material microstereolithography. Int. J. Adv. Manuf. Technol..

[50] Bártolo, P. J. *Stereolithography: materials, processes and applications*. (Springer Science & Business Media, 2011).

[51] Dudley D, Duncan WM, Slaughter J (2003). Emerging digital micromirror device (DMD) applications. In MOEMS display and imaging systems.

[52] Saleh, B. E. A., Teich, M. C. & Saleh, B. E. *Fundamentals of photonics*. **22**, (Wiley New York, 1991).

[53] Goodman, J. W. *Introduction to Fourier optics*. (Roberts and Company Publishers, 2005).

[54] Jacobs, P. F. Fundamentals of stereolithography. In *1992 International Solid Freeform Fabrication Symposium* (1992).

[55] Born, M. & Wolf, E. *Principles of optics: electromagnetic theory of propagation, interference and diffraction of light*. (Elsevier, 2013).

[56] Urrios A (2016). 3D-printing of transparent bio-microfluidic devices in PEG-DA. Lab Chip.

[57] Hua Gong ATW, High GPN (2015). density 3D printed microfluidic valves, pumps, and multiplexers. Lab Chip.

