# Publisher Correction: A compact LED-based projection microstereolithography for producing 3D microstructures

**DOI:** 10.1038/s41598-020-59557-4

**Published:** 2020-02-17

**Authors:** Ebrahim Behroodi, Hamid Latifi, Farhood Najafi

**Affiliations:** 1 Laser and Plasma Research Institute, Shahid Beheshti University, Tehran, 1983963113 Iran; 2 Department of Physics, Shahid Beheshti University, Tehran, 1983963113 Iran; 3 Department of Resin and Additives, Institute for Color Science and Technology, Tehran, 16765-654 Iran

Correction to: *Scientific Reports* 10.1038/s41598-019-56044-3, published online 23 December 2019

A supplementary file containing Figure S1 was inadvertently omitted from the original version of this Article. This error has now been corrected in the HTML and PDF versions of the Article.

