# Supplementary material for: A compact LED-based projection microstereolithography for producing 3D microstructures
Source: Sci Rep. 2019 Dec 23;9:19692. doi: 10.1038/s41598-019-56044-3 (PMC6928235; doi:10.1038/s41598-019-56044-3)
Supplement: Supplementary file 1 — Supplemental Information. [file 41598_2019_56044_MOESM1_ESM.pdf]

**Supplementary Info File**

# A compact LED-based projection microstereolithography for producing 3D microstructures

Ebrahim Behroodi[1], Hamid Latifi[1,2*], Farhood Najafi[3]

1. Laser and Plasma Research Institute, Shahid Beheshti University, Tehran 1983963113, Iran

2. Department of Physics, Shahid Beheshti University, Tehran 1983963113, Iran

3. Department of Resin and Additives, Institute for Color Science and Technology, Tehran 16765-654, Iran

* Corresponding author: latifi@sbu.ac.ir

## The effect of surface treatment

After curing, the acrylate resin did not firmly adhere to the glass surface. Therefore, after washing with isopropanol, it was easily removed from the glass surface. To solve this problem, the glass surface was treated using TMSPM based on the procedure explained in section "surface treatment". The effect of surface modification on the printed layer has been shown in Supplementary Fig. S1 (a &b).

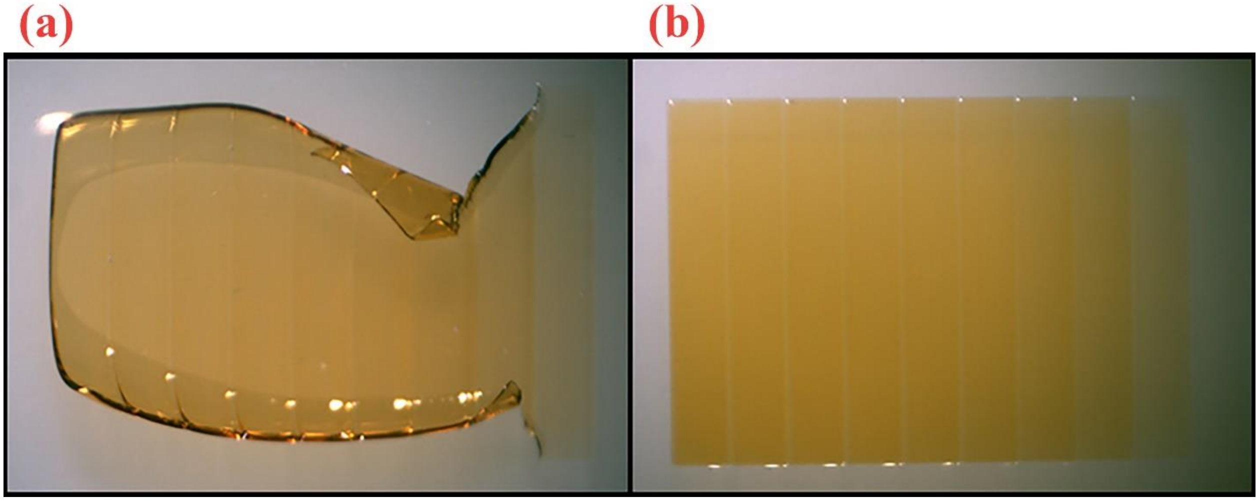


*Supplementary Fig. S1: The effect of glass surface modification* ***(a)*** *before surface treatment: the printed layer was peeled off from the glass surface and* ***(b)*** *after surface treatment: the printed layer was perfectly attached to the glass surface*
